# Homœopathic Experiments

**Published:** 1843-11

**Authors:** 


*Homœopathic Experiments.* By Andral.—(From the Bulletin Generale Therapeutiqe, May, 1834.)—Andral, at the Hopital de la Pitié, has been putting to the test of experiment the homœopathic doctrine since November. These experiments are not yet concluded, but we shall give the results as far as they go, which are quite conclusive against the views of Hahnemann. Andral treated the patients submitted to homœopathia strictly in accordance with Hahnemann's principles. The symptoms were combated by medicines, the special properties of which were pointed out by the German physician, and which, to insure the greatest exactness, were made up at the establishment of Guibourt, to which the homœopathic physicians send their patients. The regimen was carefully watched, and was altogether in accordance with that recommended by Hahnemann. It was composed of broth without salt or vegetables, of paps or of milk potages, and when the patients could eat, they were allowed bread and wine, meat which had served to make broth, and roast, rarely fish. Vegetables were never given to them, nor was any of their food seasoned. For drink they had sugar and water. During the treatment all external medication was interdicted. It is impossible, with all these precautions, not to have sufficient *data* to judge of the doctrine, especially when the facts are so numerous, and the physician so acute. The facts were collected with the most minute attention by Mr. Vernois, one of the *internes.*

In 54 applications of the homœopathic treatment, 8 patients alone derived any benefit, which continued without the use of any other plan of treatment, and 46 were as bad some days after the administration of the globules as before. It ought, however, to be mentioned that the state of 7 of them was slightly ameliorated on the morning after they took the medicine. But what were these cases? The first was a case of intercurrent pain, which had existed for some days; the second was a case of *angina;* the third, one of rheumatic pains; the fourth, intercurrent cephalalgia in a phthisical patient; the fifth was a case of stunning in a man subject to cerebral congestion; the sixth, a case of diarrhœa following constipation; the seventh, one of rheumatism at the 18th day; and eighth, a case of slight pain, which came on during chronic *gastro-enteritis.*

The medicines employed by Andral, were *aconitum, arnica, belladonna,* briony, camphor, camomile, *colchicum, ipecacuanha, hyoscyamus, opium,* soluble mercury, *nux vomica,* metallic lead, *pulsatilla nigricans,* and tin.

From the month of January, Andral treated 35 patients on homœopathic principles. Of these, 18 were men, and 17 women. Five were submitted to the *aconitum,* four to the *arnica,* five to *belladonna,* five to bryony, one to camomile, three to colchicum, three to hyoscyamus, one to opium, two to soluble mercury, three to *nux vomica,* one to lead, and two to the *pulsatilla nigricans.* We throw the results, &c., into a table. [Here follow 35 cases, with the names of the substances used, the diseases, predominant symptoms, effects, &c., in none of which, save five, was there any relief.] From the above facts, it would appear that the homœopathic plan of treating diseases is totally inert, and can be useful only as a *placebo* to hypochondriacs and nervous women, by relieving them from swallowing the manifold drugs with which they think it their duty to burden their stomachs.—*Bost. Med. and Surg. Jour.*, from *Edin. Med. and Surg. Jour.*

